# Supplementary material for: Cooking Methods and Their Relationship with Anthropometrics and Cardiovascular Risk Factors among Older Spanish Adults
Source: Nutrients. 2022 Aug 20;14(16):3426. doi: 10.3390/nu14163426 (PMC9414627; doi:10.3390/nu14163426)
Supplement: Supplementary file 1 [file nutrients-14-03426-s001.zip › nutrients-1818065-supplementary.pdf]

Supplemental Table S1. Cooking methods description.

Cooking method	Description
Raw	Neither cooked nor preserved.
Boiling	To cook in water or liquids when the temperature is at boiling point.
Roasting	To cook in an oven using hot air or radiation.
Pan-frying	To cook food in a pan with the minimum amount of oil.
Frying	To cook in hot fats or oils where the food is immersed; when the food is breaded, floured or battered and then fried.
Toasting	Browning of the food using radiant heat.
Sautéing	To cook food quickly with a few tablespoons of oil in a skillet over a medium to high heat.
Stewing	To boil slowly or simmering in a liquid for a long period of time at a low heat.

Supplemental Table S2. Adjusted means (95% confidence interval), percentage difference, and p for linear trend across quintiles of fried food consumption.

	Fried food consumption						
	Q1	Q2	Q3	Q4	Q5	PD	*p*
**n (median, g/kg of body weight) in men**	232 (0.038)	232 (0.131)	232 (0.230)	231 (0.335)	231 (0.557)		
**n (median, g/kg of body weight) in women**	262 (0.034)	262 (0.082)	261 (0.177)	262 (0.340)	261 (0.561)		
**Anthropometrics**							
**Weight (kg)**	72.0 (71.4-72.6)	73.1 (72.4-73.7)	73.1 (72.4-73.7)	72.4 (71.8-73.1)	68.9 (68.2-69.5)	-4.30	<0.001
**BMI (kg/m2)**	27.7 (27.5-57.8)	28.0 (27.8-28.2)	28.1 (27.9-28.2)	27.9 (27.8-28.1)	27.0 (26.8-27.1)	-2.50	<0.001
**MUAC (cm)**	28.7 (28.6-28.8)	29.1 (29.0-29.2)	29.0 (28.9-29.1)	28.6 (28.5-28.7)	27.6 (27.5-27.7)	-3.80	<0.001
**Waist circumference (cm)**	95.4 (94.8-95.9)	96.6 (96.1-97.1)	97.1 (96.5-97.6)	96.4 (95.8-97.0)	94.2 (93.7-94.8)	-1.30	<0.001
**Hip circumference (cm)**	103.5 (103.2-103.8)	103.5 (103.2-103.8)	103.9 (103.6-104.2)	103.1 (102.8-103.4)	100.7 (100.3-101)	-2.70	<0.001
**Calf circumference (cm)**	34.2 (34.1-34.3)	34.3 (34.2-34.4)	34.2 (34.1-34.3)	33.6 (33.5-33.7)	32.7 (32.6-32.8)	-4.40	<0.001
**Cardiovascular risk factors**							
**Total cholesterol (mg/dL)**	192.1 (190.1-193.6)	188.4 (187.0-189.9)	188.4 (186.9-189.9)	190.4 (188.9-191.9)	190.5 (189.2-191.9)	-0.80	0.671
**HDL-cholesterol (mg/dL)**	54.4 (53.8-55.0)	53.1 (52.5-53.7)	53.8 (53.2-54.4)	54.0 (53.4-54.6)	55.0 (54.4-55.6)	1.10	0.201
**LDL-cholesterol (mg/dL)**	115.6 (114.4-116.8)	113.1 (112.0-114.2)	112.3 (111.2-113.5)	113.9 (112.8-115.0)	113.2 (112.2-114.2)	-2.10	0.273
**Triglycerides (mg/dL)**	103.3 (102.4-104.1)	105.2 (104.3-106.2)	104.3 (103.4-105.2)	105.3 (104.4-106.2)	104.3 (103.4-105.2)	1.00	0.707
**Glucose (mg/dL)**	96.3 (95.7-96.9)	98.2 (97.6-98.8)	96.5 (95.9-97.1)	99.0 (98.4-99.6)	98.2 (97.6-98.7)	2.00	0.665
**HbA1c (%)**	5.71 (5.69-5.74)	5.81 (5.78-5.83)	5.78 (5.76-5.81)	5.76 (5.73-5.79)	5.88 (5.85-5.91)	3.00	0.176
**Insulin (µU/mL)**	9.47 (9.25-9.69)	10.4 (10.1-106)	9.81 (9.55-10.1)	10.2 (9.94-10.4)	9.45 (9.17-9.74)	-0.20	0.215
**Blood pressure**							
**Casual SBP (mmHg)**	135.6 (135.4-135.8)	134.3 (134.1-134.5)	135.5 135.3-135.7)	136.2 (136.0-136.4)	136.4 (136.2-136.6)	0.60	0.525
**Casual DBP (mmHg)**	79.6 (79.3-79.9)	79.3 (79.0-79.5)	80.2 (80.0-80.8)	80.6 (80.3-80.8)	80.4 (80.1-80.7)	1.00	0.354
**Casual HR (bpm)**	70.0 (69.8-70.3)	70.3 (70.0-70.5)	69.9 (69.7-70.1)	70.7 (70.5-70.9)	70.0 (69.7-70.2)	0.00	0.819
**24 h-SBP (mmHg)**	126.0 (125.8-126.1)	126.3 (126.1-126.4)	127.5 (127.3-127.6)	127.8 (127.6-128.0)	128.0 (127.9-128.2)	1.60	0.044
**24 h-DBP (mmHg)**	73.8 (73.6-74.0)	74.2 (74.0-74.4)	74.7 (74.5-75.0)	74.6 (74.3-74.8)	75.0 (74.7-75.2)	1.60	0.103
**24 h-HR (bpm)**	67.4 (67.1-67.6)	67.8 (67.6-68.0)	67.7 (67.5-68.0)	68.6 (68.3-68.8)	68.5 (68.2-68.7)	1.60	0.056
**Cardiac function biomarkers**							
**NT-proBNP (pg/mL)**	87.0 (84.6-89.4)	79.6 (77.5-81.7)	87.6 (85.4-89.9)	87.1 (84.9-89.3)	90.8 (88.5-93.3)	4.30	0.150
**Troponin T (ng/L)**	9.40 (9.20-9.60)	9.30 (9.10-9.50)	9.70 (9.50-9.90)	9.80 (9.50-9.90)	10.0 (9.80-10.2)	6.40	0.077

Analyses were adjusted for sex, age (continuous), educational level (primary or less, secondary, university), cigarette smoking status (former, current, never), alcohol consumption (continuous), ex-drinker status (yes, no), recreational physical activity in METS·hours/week (continuous), household physical activity in METS·hours/week (continuous), hours of television (continuous), number of chronic diseases (continuous), number of prescribed medications (continuous), energy intake in Kcal/d (continuous), very long chain omega-3 fatty acids consumption (continuous), and fiber consumption (continuous).

PDs: percentage difference calculated as [(5th quintile – 1st quintile)/ 1st quintile] x 100, p: p for linear trend. BMI: Body Mass Index, MUAC: Mid-Upper Arm Circumference, SBP: Systolic Blood Pressure, DBP: Diastolic Blood Pressure, HR: Heart rate. Analyses for HbA1c and insulin were performed in a nested subsample of 1,066 participants

Supplemental Table S3. Adjusted means (95% confidence interval), percentage difference, and p for linear trend across quintiles of toasted food consumption.

	Toasted food consumption						
	Q1	Q2	Q3	Q4	Q5	PD	*p*
**n (median, g/kg of body weight) in men**	340 (0.00)	124 (0.140)	232 (0.529)	232 (0.900)	231 (1.40)		
**n (median, g/kg of body weight) in women**	293 (0.00)	231 (0.016)	231 (0.540)	262 (0.914)	261 (1.31)		
**Anthropometrics**							
**Weight (kg)**	73.1 (72.5-73.6)	72.3 (71.6-73.0)	73.6 (72.9-74.2)	72.8 (72.2-73.4)	67.5 (66.9-68.1)	-7.70	<0.001
**BMI (kg/m2)**	28.2 (28.0-28.3)	27.2 (28.11+28.4)	28.3 (28.2-28.5)	27.8 (27.7-28.0)	26.1 (26.0-26.2)	-7.60	<0.001
**MUAC (cm)**	28.5 (28.4-28.6)	28.6 (28.5-28.7)	28.9 (28.9-29.0)	29.1 (29.0-29.1)	28.0 (27.9-28.1)	-1.80	0.448
**Waist circumference (cm)**	97.2 (96.7-97.7)	96.0 (95.4-96.5)	97.3 (96.4-97.4)	96.9 (96.4-97.4)	92.0 (91.4-92.5)	-5.40	<0.001
**Hip circumference (cm)**	102.9 (102.6-103.2)	104.1 (103.8-104.5)	104.3 (104.0-104.6)	03.4 (103.1-103.7)	100.2 (99.9-100.5)	-2.60	<0.001
**Calf circumference (cm)**	33.7 (33.6-33.8)	33.8 (33.7-33.9)	34.2 (34.1-34.3)	34.2 (34.1-34.2)	33.2 (33.1-33.3)	-1.50	0.070
**Cardiovascular risk factors**							
**Total cholesterol (mg/dL)**	186.0 (184.7-187.3)	193.2 (191.6-194.8)	189.3 (187.9-190.7)	90.8 (189.4-192.3)	192.6 (191.1-194.1)	3.60	0.647
**HDL-cholesterol (mg/dL)**	53.5 (52.9-54.0)	55.1 (54.4-55.7)	53.0 (52.5-53.6)	53.9 (53.3-54.5)	55.4 (54.8-55.9)	3.60	0.863
**LDL-cholesterol (mg/dL)**	109.8 (108.8-110.8)	115.6 (114.3-116.8)	113.8 (112.7-114.8)	14.2 (113.1-115.2)	116.5 (115.4-117.5)	6.10	0.252
**Triglycerides (mg/dL)**	106.5 (105.6-107.3)	105.9 (104.9-106.9)	105.7 (104.7-106.7)	06.6 (105.8-107.5)	97.7 (96.9-98.5)	-8.30	0.119
**Glucose (mg/dL)**	100.8 (100.3-101.4)	97.1 (96.5-97.7)	97.1 (96.597.7)	98.7 (98.1-99.3)	93.4 (93.0-94.1)	-7.30	0.002
**HbA1c (%)**	5.84 (5.81-5.87)	5.74 (5.71-5.78)	5.33 (5.71-5.76)	5.83 (5.81-5.86)	5.75 (5.72-5.78)	-1.50	0.544
**Insulin (µU/mL)**	9.80 (9.54-10.1)	10.7 (10.4-11.1)	9.87 (9.61-10.1)	10.1 (9.88-10.3)	9.22 (9.01-9.44)	-5.90	0.602
**Blood pressure**							
**Casual SBP (mmHg)**	136.7 (136.5-136.9)	136.2 (135.9-136.4)	135.2 (135.0-135.4)	35.3 (135.1-135.5)	134.4 (134.2-134.6)	-1.70	0.045
**Casual DBP (mmHg)**	80.7 (80.4-80.9)	80.2 (79.9-80.5)	79.9 (79.7-80.2)	79.7 (79.4-80.0)	79.4 (79.1-79.6)	-1.60	0.019
**Casual HR (bpm)**	70.8 (70.6-71.0)	70.9 (70.6-71.2)	69.8 (69.6-70.1)	70.3 (70.0-70.5)	69.1 (68.9-69.3)	-2.40	0.040
**24 h-SBP (mmHg)**	128.5 (128.4-128.7)	128.2 (128.0-128.4)	126.5 (126.3-126.7)	27.2 (127.0-127.3)	125.1 (124.9-125.2)	-2.70	0.001
**24 h-DBP (mmHg)**	75.2 (74.9-75.4)	74.5 (74.3-7.8)	74.4 (74.2-74.6)	74.4 (74.1-74.6)	73.6 (73.4-73.8)	-2.10	<0.001
**24 h-HR (bpm)**	68.4 (68.2-68.6)	68.7 (68.5-69.0)	68.1 (67.8-68.3)	68.2 (68.0-68.4)	66.6 (66.4-66.8)	-2.60	0.017
**Cardiac function biomarkers**							
**NT-proBNP (pg/mL)**	90.3 (88.1-92.5)	93.3 (90.4-96.3)	83.2 (81.1-85.3)	79.9 (77.9-82.0)	86.5 (84.3-88.7)	-4.20	0.321
**Troponin T (ng/L)**	10.0 (9.80-10.1)	9.60 (9.40-9.90)	9.80 (9.60-10.0)	9.40 (9.20-9.60)	9.20 (9.00-9.40)	-8.00	0.388

Analyses were adjusted for sex, age (continuous), educational level (primary or less, secondary, university), cigarette smoking status (former, current, never), alcohol consumption (continuous), ex-drinker status (yes, no), recreational physical activity in METS·hours/week (continuous), household physical activity in METS·hours/week (continuous), hours of television (continuous), number of chronic diseases (continuous), number of prescribed medications (continuous), energy intake in Kcal/d (continuous), very long chain omega-3 fatty acids consumption (continuous), and fiber consumption (continuous).

PDs: percentage difference calculated as [(5th quintile – 1st quintile)/ 1st quintile] x 100, p: p for linear trend. BMI: Body Mass Index, MUAC: Mid-Upper Arm Circumference, SBP: Systolic Blood Pressure, DBP: Diastolic Blood Pressure, HR: Heart rate. Analyses for HbA1c and insulin were performed in a nested subsample of 1,066 participants.

Supplemental Table S4. Adjusted means (95% confidence interval), percentage difference, and p for linear trend across quintiles of sautéed food consumption.

	Sautéed food consumption						
	**Q1**	**Q2**	**Q3**	**Q4**	**Q5**	**PD**	**p**
**n (median, g/kg of body weight) in men**	232 (0.042)	232 (0.154)	232 (0.261)	232 (0.373)	231 (0.603)		
**n (median, g/kg of body weight) in women**	262 (0.029)	262 (0.152)	261 (0.260)	262 (0.371)	261 (0.618)		
**Anthropometrics**							
**Weight (kg)**	72.6 (72.0-73.2)	73.0 (72.4-73.6)	72.7 (72.1-73.3)	71.4 (70.8-72.0)	69.7 (69.0-80.3)	-4.00	<0.001
**BMI (kg/m2)**	27.8 (27.7-28.0)	28.0 (27.9-28.2)	28.0 (27.8-28.1)	27.6 (27.5-27.8)	27.2 (27.0-27.3)	-2.20	0.002
**MUAC (cm)**	28.7 (28.6-28.8)	28.7 (28.6-28.8)	28.8 (28.7-28.9)	28.6 (28.6-28.7)	28.2 (28.1-28.2)	-1.70	0.008
**Waist circumference (cm)**	96.3 (95.8-96.8)	96.7 (96.1-97.2)	96.6 (96.1-97.2)	95.6 (95.1-96.1)	94.5 (93.9-95.0)	-1.90	0.001
**Hip circumference (cm)**	103.0 (102.7-103.3)	103.3 (103.0-103.6)	103.7 (103.4-104.0)	103.2 (102.9-103.5)	101.5 (101.2-101.8)	-1.50	0.010
**Calf circumference (cm)**	33.8 (33.8-33.9)	33.9(33.8-34.0)	34.0 (33.9-34.1)	33.8 (33.8-33.9)	33.4 (33.3-33.4)	-1.20	0.015
**Cardiovascular risk factors**							
**Total cholesterol (mg/dL)**	189.9 (188.4-191.4)	188.5 (187.0-190.0)	190.7 (189.3-192.1)	189.8 (188.4-191.2)	191.1 (189.5-192.5)	0.60	0.633
**HDL-cholesterol (mg/dL)**	55.1 (54.5-55.7)	53.7 (53.1-54.3)	53.6 (53.0-24.3)	53.8 (53.3-54.4)	54.1 (53.5-54.7)	-1.80	0.363
**LDL-cholesterol (mg/dL)**	112.8 (111.7-113.9)	111.7 (110.6-112.9)	114.8 (113.7-115.9)	114.4 (113.3-115.5)	114.5 (113.4-115.5)	1.50	0.239
**Triglycerides (mg/dL)**	103.4 (120.5-104.4)	109.0 (108.1-110.0)	140.3 (103.4-105.2)	101.1 (100.3-102.0)	104.5 (103.6-105.4)	1.10	0.248
**Glucose (mg/dL)**	96.1 (95.5-96.7)	99.0 (98.4-99.5)	97.9 (97.3-98.5)	97.4 (96.8-97.9)	97.9 (97.3-98.5)	1.90	0.360
**HbA1c (%)**	5.73 (5.71-5.76)	5.83 (5.80-5.86)	5.81 (5.79-5.84)	5.77 (5.74-5.79)	5.78 (5.75-5.81)	0.90	0.970
**Insulin (μU/mL)**	9.89 (9.65-10.1)	9.95 (9.68-10.2)	10.3 (10.1-10.6)	9.58 (9.34-9.82)	9.62 (9.37-9.86)	-2.70	0.192
**Blood pressure**							
**Casual SBP (mmHg)**	135.9 (135.7-136.1)	136.2 (136.0-136.4)	135.3 (135.1-135.5)	136.0 (135.8-136.2)	135.7 (134.5-134.9)	-0.20	0.325
**Casual DBP (mmHg)**	80.2 (79.9-80.4)	80.5 (80.2-80.8)	80.3 (80.0-80.6)	79.7(79.4-80.0)	79.3 (79.0-79.6)	-1.10	0.047
**Casual HR (bpm)**	69.4 (69.1-69.6)	70.9 (70.7-71.2)	70.1 (69.9-70.3)	69.8 (69.5-70.0)	70.7 (70.4-70.9)	1.90	0.641
**24 h-SBP (mmHg)**	127.5 (127.4-127.7)	127.5 (127.3-127.7)	127.0 (126.9-127.2)	126.7 (126.6-126.9)	126.7 (126.52-126.9)	-0.60	0.168
**24 h-DBP (mmHg)**	74.6 (74.4-74.8)	74.7 (74.5-75.0)	74.7 (74.4-74.9)	74.2 (73.9-74.4)	74.1 (73.9-74.4)	-0.70	0.088
**24 h-HR (bpm)**	67.7 (67.5-67.9)	68.3 (68.1-68.5)	68.1 (67.9-68.4)	67.7 (67.4-67.9)	68.1 (67.9-68.3)	0.70	0.653
**Cardiac function biomarkers**							
**NT-proBNP (pg/mL)**	88.3 (86.0-90.6)	93.5 (91.0-96.1)	79.4 (77.3-81.6)	85.8 (83.6-87.9)	85.3 (83.2-87.6)	-3.40	0.437
**Troponin T (ng/L)**	9.80 (9.60-10.0)	10.2 (10.0-10.4)	9.40 (9.20-9.60)	9.40 (9.20-9.60)	9.30 (9.10-9.50)	-5.10	0.007

Analyses were adjusted for sex, age (continuous), educational level (primary or less, secondary, university), cigarette smoking status (former, current, never), alcohol consumption (continuous), ex-drinker status (yes, no), recreational physical activity in METS·hours/week (continuous), household physical activity in METS·hours/week (continuous), hours of television (continuous), number of chronic diseases (continuous), number of prescribed medications (continuous), energy intake in Kcal/d (continuous), very long chain omega-3 fatty acids consumption (continuous), and fiber consumption (continuous).

PDs: percentage difference calculated as [(5th quintile – 1st quintile)/ 1st quintile] x 100, p: p for linear trend. BMI: Body Mass Index, MUAC: Mid-Upper Arm Circumference, SBP: Systolic Blood Pressure, DBP: Diastolic Blood Pressure, HR: Heart rate. Analyses for HbA1c and insulin were performed in a nested subsample of 1,066 participants.

Supplemental Table S5. Adjusted means (95% confidence interval), percentage difference, and p for linear trend across quintiles of stewed food consumption.

	Stewed food consumption						
	Q1	Q2	Q3	Q4	Q5	PD	p
**n (median, g/kg of body weight) in men**	232 (0.000)	232 (0.054)	232 (0.167)	232 (0.321)	231 (0.604)		
**n (median, g/kg of body weight) in women**	262 (0.000)	262 (0.081)	261 (0.165)	262 (0.336)	261 (0.647)		
**Anthropometrics**							
**Weight (kg)**	73.5 (72.9-74.2)	73.4 (72.8-74.0)	71.1 (70.5-71.8)	71.9 /71.3-72.6)	69.4 (68.8-70.0)	-5.60	<0.001
**BMI (kg/m2)***	28.3 (28.1-28.5)	28.2 (28.0-28.3)	27.5 (27.4-27.7)	27.6 (27.5-27.8)	27.0 (26.8-27.2)	-4.60	<0.001
**MUAC (cm)***	28.8 (28.7-28.9)	28.8 (28.7-28.9)	28.4 (28.3-28.5)	28.4 (28.3-28.5)	28.6 (28.5-28.7)	-0.70	0.002
**Waist circumference (cm)**	96.7 (96.2-97.3)	97.5 (96.9-98.0)	95.4 (94.9-96.0)	96.1 (95.6-96.7)	93.9 (93.4-94.4)	-2.90	<0.001
**Hip circumference (cm)**	104.1 (103.8-104.4)	103.6 (103.3-103.9)	102.8 (102.5-103.1)	102.7 (102.5-103.0)	101.4 (101.1-101.7)	-2.60	<0.001
**Calf circumference (cm)**	33.8 (33.8-33.9)	33.9 (33.8-34.0)	33.6 (33.5-33.7)	33.8 (33.7-33.9)	33.8 (33.7-33.9)	0.00	0.084
**Cardiovascular risk factors**							
**Total cholesterol (mg/dL)**	188.4 (186.9-189.9)	189.2 (187.7-190.7)	190.2 (188.7-191.7)	189.5 (188.0-191.0)	192.5 (191.2-192.9)	2.20	0.384
**HDL-cholesterol (mg/dL)**	54.1 (53.5-54.7)	53.2 (52.6-53.7)	54.1 (53.5-54.7)	53.9 (53.3-54.4)	55.1 (54.6-55.7)	1.80	0.145
**LDL-cholesterol (mg/dL)**	111.6 (110.5-112.8)	113.7 (112.6-114.8)	114.4 (113.3-115.5)	112.8 (111.7-113.9)	115.6 (114.6-116.6)	3.60	0.436
**Triglycerides (mg/dL)**	106.2 (105.3-107.2)	104.8 (103.8-105.7)	102.7 (101.8-103.6)	106.7 (105.7-107.6)	102.1 (101.2-103.0)	-3.90	0.160
**Glucose (mg/dL)**	97.9 (97.3-98.5)	98.7 (98.2-99.3)	97.4 (96.8-98.0)	97.3 (96.7-97.9)	96.9 (96.3-97.4)	-1.00	0.239
**HbA1c (%)**	5.81 (5.78-5.84)	5.80 (5.77-5.83)	5.78 (5.75-5.80)	5.77 (8.75-5.80)	5.75 (5.72-5.77)	-1.00	0.276
**Insulin (µU/mL)**	10.2 (9.96-10.5)	9.94 (9.69-10.2)	9.96 (9.71-10.2)	9.55 (9.32-9.79)	9.63 (9.37-9.89)	-5.60	0.038
**Blood pressure**							
**Casual SBP (mmHg)**	136.4 (136.2-136.6)	135.5 (135.3-135.7)	135.1 (134.9-135.3)	135.2 (135.0-135.4)	135.8 (135.6-136.0)	-0.40	0.514
**Casual DBP (mmHg)**	79.8 (79.5-80.1)	80.0 (79.7-80.2)	80.5 (80.2-80.8)	79.8 (79.5-80.0)	80.0 (79.7-80.3)	0.30	0.447
**Casual HR (bpm)**	70.9 (70.7-71.1)	69.7 (69.4-69.9)	70.5 (70.2-70.7)	69.7 (69.5-70.0)	70.0 (69.8-70.3)	-1.30	0.100
**24 h-SBP (mmHg)**	127.6 (127.4-127.8)	127.9 (127.7-128.1)	127.1 (126.9-127.3)	126.4 (126.2-126.6)	126.5 (126.4-126.7)	-0.90	0.039
**24 h-DBP (mmHg)**	74.4 (74.2-74.7)	74.6 (74.4-74.9)	74.5 (74.3-74.8)	74.3 (74.0-74.5)	74.4 (74.2-74.6)	0.00	0.261
**24 h-HR (bpm)**	68.4 (68.1-68.6)	67.7 (67.4-67.9)	68.1 (67.9-68.3)	67.9(67.7-68.1)	67.9 (67.7-68.1)	-0.70	0.313
**Cardiac function biomarkers**							
**NT-proBNP (pg/mL)**	88.8 (86.4-91.2)	87.5 (85.2-89.8)	87.9 (85.6-90.3)	85.6 (83.4-87.8)	82.1 (80.0-84.3)	-7.50	0.521
**Troponin T (ng/L)**	9.80 (9.60-10.0)	9.70 (9.50-9.90)	10.1 (9.90-10.3)	9.40 (9.20-9.60)	9.20 (9.00-9.40)	-6.10	0.078

Analyses were adjusted for sex, age (continuous), educational level (primary or less, secondary, university), cigarette smoking status (former, current, never), alcohol consumption (continuous), ex-drinker status (yes, no), recreational physical activity in METS·hours/week (continuous), household physical activity in METS·hours/week (continuous), hours of television (continuous), number of chronic diseases (continuous), number of prescribed medications (continuous), energy intake in Kcal/d (continuous), very long chain omega-3 fatty acids consumption (continuous), and fiber consumption (continuous).

PDs: percentage difference calculated as [(5th quintile – 1st quintile)/ 1st quintile] x 100, p: p for linear trend. BMI: Body Mass Index, MUAC: Mid-Upper Arm Circumference, SBP: Systolic Blood Pressure, DBP: Diastolic Blood Pressure, HR: Heart rate. Analyses for HbA1c and insulin were performed in a nested subsample of 1,066 participants.

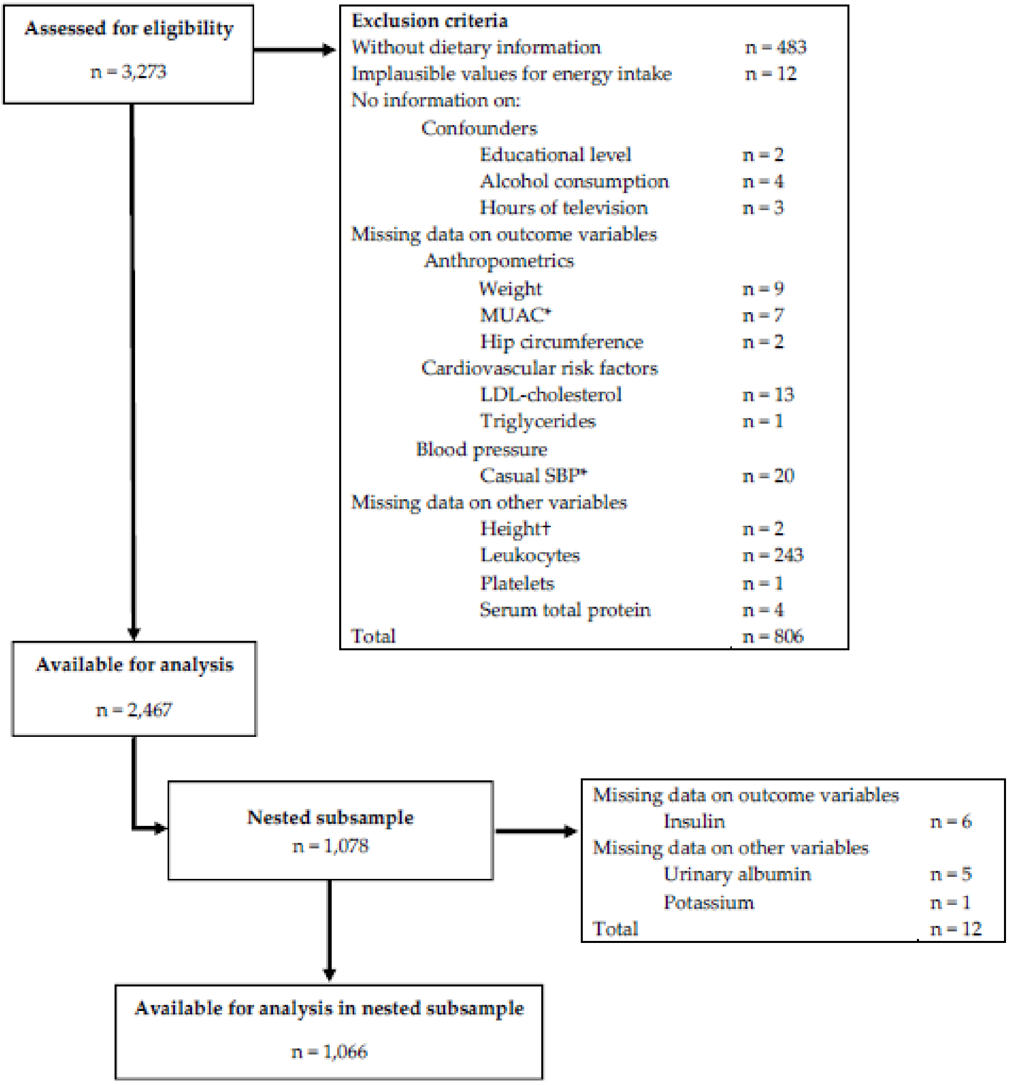

†Height used to calculate Body Mass Index (BMI).

*MUAC: Mid-Upper Arm Circumference, SBP: systolic blood pressure.

Supplemental Figure S1: Flow chart
